# Procedure for the Isolation of Endothelial Cells from Human Cerebral Arteriovenous Malformation (cAVM) Tissues

**DOI:** 10.3389/fncel.2018.00030

**Published:** 2018-02-07

**Authors:** Qiang Hao, Xiao-Lin Chen, Li Ma, Tong-Tong Wang, Yue Hu, Yuan-Li Zhao

**Affiliations:** ^1^Department of Neurosurgery, Beijing Tiantan Hospital, Capital Medical University, Beijing, China; ^2^Department of Neurosurgery, Peking University International Hospital, Peking University, Beijing, China; ^3^Basic Medical Science Department, Capital Medical University, Beijing, China; ^4^China National Clinical Research Center for Neurological Diseases, Beijing, China; ^5^Stroke Center, Beijing Institute for Brain Disorders, Beijing, China; ^6^Beijing Key Laboratory of Translational Medicine for Cerebrovascular Disease, Beijing, China

**Keywords:** endothelial cells, cerebral arteriovenous malformation, isolation approach, purification, angiogenesis, *in vitro* culture

## Abstract

In this study, we successfully established a stable method for the isolation of endothelial cells (ECs) from human cerebral arteriovenous malformation (cAVM) tissues. Despite human cAVM tissues having a minor population of ECs, they play an important role in the manifestation and development of cAVM as well as in hemorrhagic stroke and thrombogenesis. To characterize and understand the biology of ECs in human cAVM (cAVM-ECs), methods for the isolation and purification of these cells are necessary. We have developed this method to reliably obtain pure populations of ECs from cAVMs. To obtain pure cell populations, cAVM tissues were mechanically and enzymatically digested and the resulting single cAVM-ECs suspensions were then labeled with antibodies of specific cell antigens and selected by flow cytometry. Purified ECs were detected using specific makers of ECs by immunostaining and used to study different cellular mechanisms. Compared to the different methods of isolating ECs from tissues, we could isolate ECs from cAVMs confidently, and the numbers of cAVM-ECs harvested were almost similar to the amounts present in vessel components. In addition to optimizing the protocol for isolation of ECs from human cAVM tissues, the protocol could also be applied to isolate ECs from other human neurovascular-diseased tissues. Depending on the tissues, the whole procedure could be completed in about 20 days.

## Introduction

**FIGURE 1:**
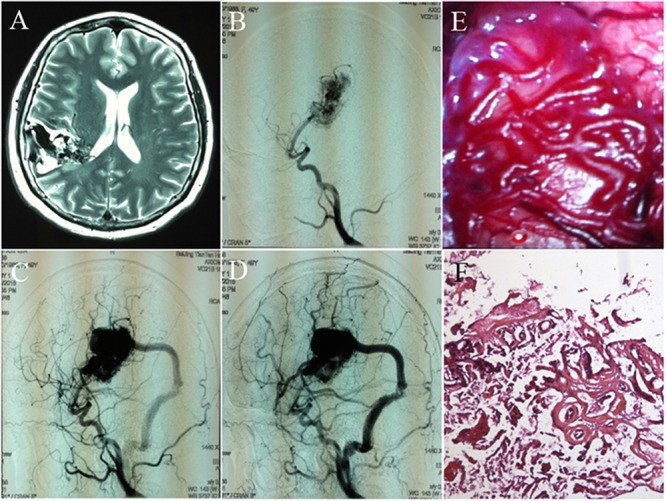
Images and histology of cerebral arteriovenous malformation (cAVM). **(A)** T2WI of MRI imaging; **(B–D)** digital subtraction angiography (DSA) showed cerebral middle artery as the feeder artery provided blood to the cAVM nidus, which connected to the sigmoid sinus through a thick draining vessel; **(E)** morphology of cAVM viewed under the operational microscope; **(F)** photomicrograph of cAVM with deformed vascular walls from the nidus of the AVM. It had numerous collagen fibers, lacked smooth muscle and elastic fibers with incomplete wall demarcations.

Cerebral arteriovenous malformations (cAVMs) are vascular lesions characterized by abnormal arteries and venous entanglement, which divert blood directly from the arteries to the venous circulation, instead of capillaries. cAVMs are rare and occur sporadically, which may be associated with genetic disorders (Consoli et al., 2013; Novakovic et al., 2013; Gross and Du, 2014; Young et al., 2015; **Figure 1**). The pathogenic mechanisms underlying cAVMs development are unknown. Currently, highly invasive procedures are the main medical treatment options for this vascular disease. However, the surgical access to cAVMs nidus can result in serious damage to adjacent brain areas and irreversible loss of neurological function. Therefore, alternative therapeutic strategies, which are safer and more efficient, are essential (Ferreira et al., 2014).

Angiogenesis is the development of new blood vessels, and is a feature in different pathologies, such as atherosclerosis, cancer, arthritis, and particularly vascular diseases (Folkman, 1995; Griffioen and Molema, 2000). To understand the manifestations of these vascular diseases, a proper understanding of ECs biology is necessary. Hence, a method to isolate and purify ECs from cAVM tissues is essential.

**FIGURE 2:**
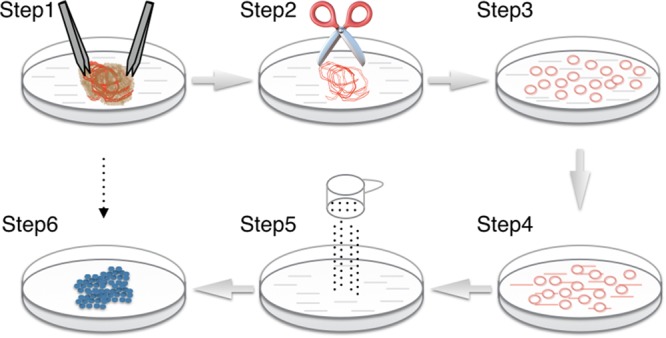
Procedure for cAVM-EC isolation from cAVM lesioned tissues. (Step 1) Separate brain tissue from surrounding vessels; (Steps 2–4) AVMs were cut into small cubes and digested using 0.25% Trypsin-EDTA; (Step 5) filter cells; (Step 6) incubated cells for 7–14 days until colonies appeared.

**FIGURE 3:**
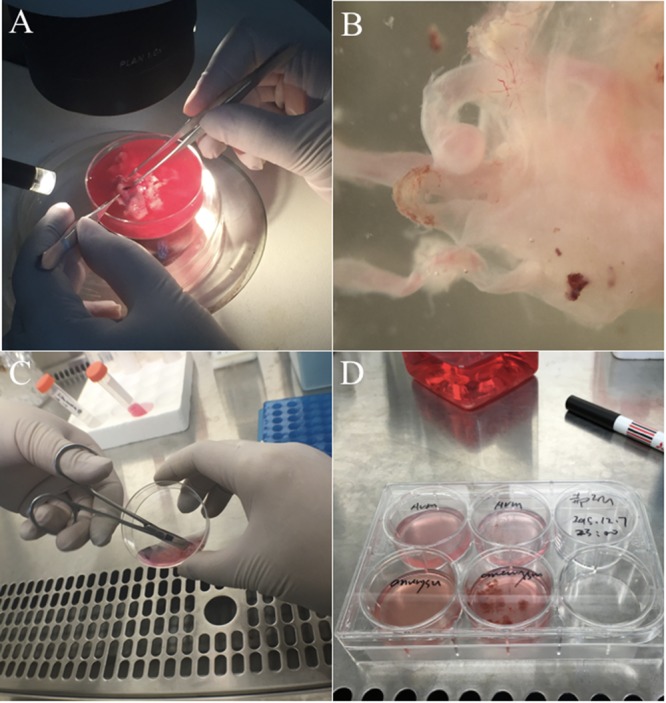
Experimental procedure to isolate cAVM-ECs. **(A)** Original cAVM lesioned tissues were washed with Phosphate-buffered saline (PBS); **(B)** the disordered vessels in the cAVM nidus, showing thin and lucid vessel layers; **(C)** disordered vessels in the cAVM nidus were cut into small cubes and digested in Trypsin-EDTA; **(D)** The isolated cells were cultured in conditional medium.

Endothelial cells express a number of specific cell surface markers, including CD31, CD34, and von Willebrand factor (vWF), which have been used to isolate, purify and identify ECs in complex cell populations (Rakocevic et al., 2017). Using this method, isolation of ECs from cAVM (cAVM-ECs) tissues was carried out by creating a single-cell suspension of cAVMs through mechanical and biological digestion. These single-cell suspensions were then labeled with antibodies and isolated and purified through FACS (**Figures 2, 3**). The purified cAVM-ECs could be used for different downstream applications, i.e., molecular profiling, genomic analysis, micro RNA analysis, proteomics, as well as for tissue engineering (Levenberg et al., 2010).

### AVM-ECs Isolation and Culture

Endothelial cells accounts for about 1–2% of the total cells in cAVMs. They are located in the inner vessel cavity and surrounded by numerous cell types (Griffioen and Molema, 2000). Thus, ECs need to be purified before they can be used for further study. There are numerous methods for the isolation of ECs from the human brain (Baev and Awad, 1998; Bernas et al., 2010; Navone et al., 2013; Zhang et al., 2013). However, we compared the different methods, and found: (1) based on Professor Zhang’ protocol, we could not harvest ECs from cAVMs reproducibly, and were only successful for 2 out of the 10 specimens; (2) we injected 0.25% Trypsin-EDTA into the cAVM nidus using a 1 ml injection syringe to digest the ECs, but the cells harvested were fibroblast-like cells and no ECs were found; (3) using the method established by us, we were able to confidently isolate ECs from cAVMs and passage them for 3–4 generations stably. These procedures included mincing and digestion, filtration, manual weeding, immunostaining, and isopycnic centrifugation of primary cultures or immortalized ECs.

### Experimental Protocol

We exploited previously published methods for the isolation and passaging of ECs from normal human cerebral tissues and neurovascular tissues (Baev and Awad, 1998; Bernas et al., 2010; Navone et al., 2013; Zhang et al., 2013). This protocol used endothelial cell medium (ECM, ScienCell, United States), which could facilitate ECs to adhere to surfaces coated by collagen type I and result in a certain proportion of ECs adhering, compared to other cell types from cAVMs. The isolated cAVM-ECs were viable and could proliferate for an extended period. Even when cultured for more than 20 days *in vitro*, cAVM-ECs maintained their endothelial features and did not undergo fibroblast *trans*-differentiation. In addition, cAVM-ECs maintained high viability and were similar to thawed and cultured cryopreserved ECs.

### Characterization of Isolated cAVM-ECs

**FIGURE 4:**
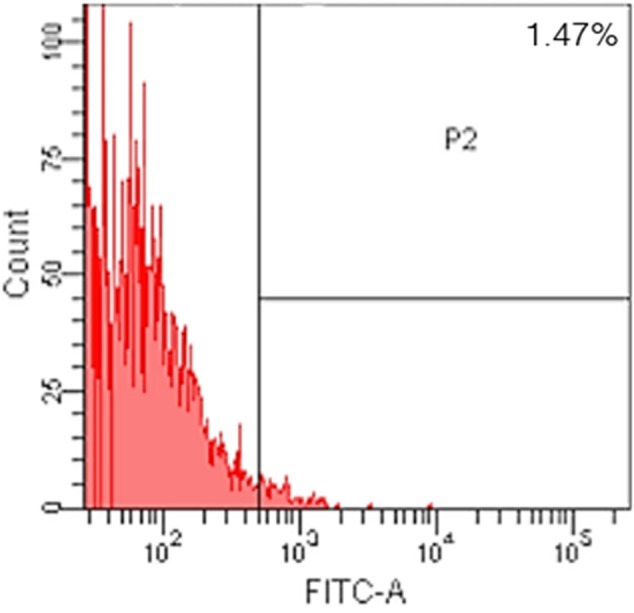
Flow cytometry analysis of cAVM-ECs. Expression of CD31 in isolated human cAVM-ECs.

**FIGURE 5:**
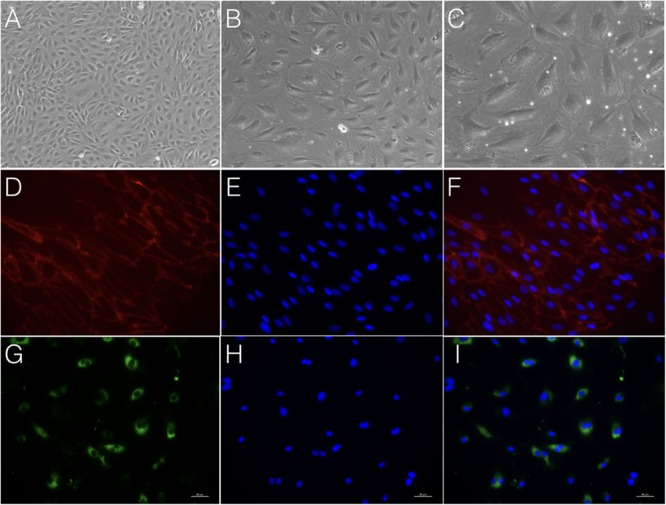
Morphology and immunostaining of cAVM-ECs. **(A–C)** morphology of cAVM-ECs in different fields of view; **(A)** scale bar = 100 μm; **(B)** scale bar = 50 μm; **(C)** scale bar = 20 μm; **(D)** CD31 expression; **(E)** 4′, 6-diamidino-2-phenylindole (DAPI) staining; **(F)** Merge, scale bar = 50 μm; **(G)** CD34 expression; **(H)** DAPI staining; **(I)** Merge, scale bar = 50 μm.

After following our isolation and purification protocol, we verified the purity of the ECs. Specific endothelial cell markers were available to identify cells of endothelial origin, like vWF, CD31, and CD34 (van Beijnum et al., 2008; Rakocevic et al., 2017). Primary ECs were sorted by FACS for CD31 positive expression. The proportion of cAVM-ECs was estimated to be around (1.53 ± 0.13)% (**Figure 4**). cAVM-ECs were sorted before they reached four passages in cell culture. Under a phase-contrast microscope, the isolated cAVM-ECs were observed to have a classic round border and cobblestone appearance in morphology and displayed a flat ellipse shape when confluent with each other (**Figures 5A–C**).

**FIGURE 6:**
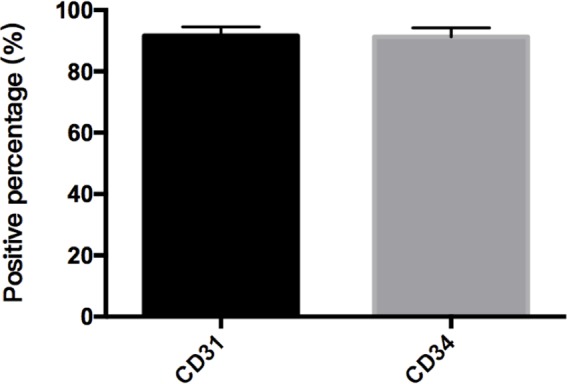
Percent of CD31 and CD34 cells in cAVM-ECs. cAVM-ECs selected by FACS were immunostained for CD31 and CD34 antigens, respectively. The percent of CD31 positive cells was about (91.6 ± 2.86), and about (91.2 ± 2.96) for CD34.

**FIGURE 7:**
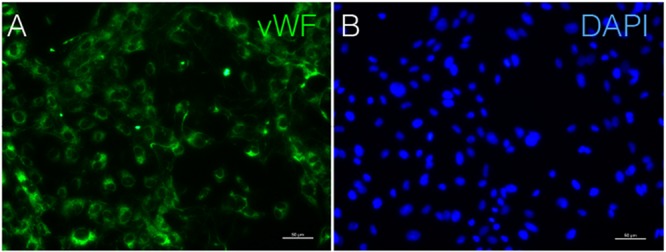
Immunostaining of vWF in cAVM-ECs. **(A)** vWF expression; **(B)** DAPI staining, scale bar = 50 μm.

**FIGURE 8:**
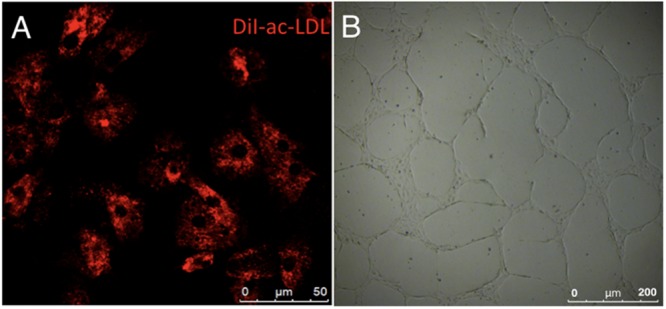
Functional phenotypes of isolated human cAVM-ECs. **(A)** LDL uptake by human cAVM-ECs, scale bar = 50 μm; **(B)** Capillary tube-like structure formation by cAVM-ECs, scale bar = 200 μm.

**FIGURE 9:**
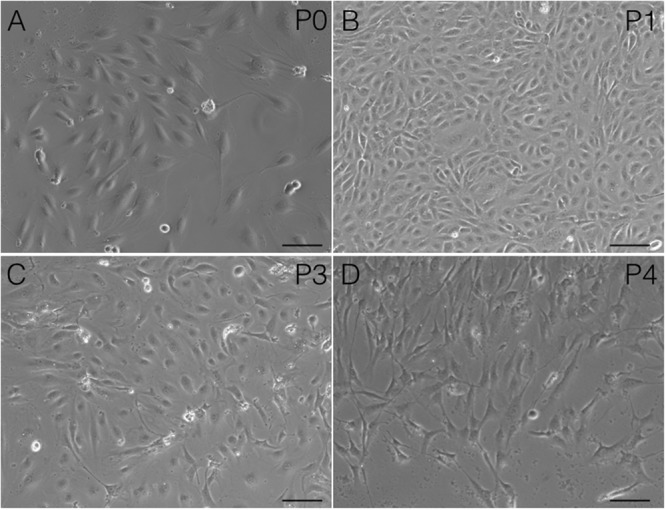
Changes in cAVM-EC morphology when isolated and passaged. **(A)** morphology in P0; **(B)** morphology in P1; **(C)** morphology in P2; **(D)** morphology in P3. Scale bar = 50 μm

Immunostaining demonstrated that more than 90% of cAVM-ECs expressed the classic EC markers, CD31 (91.6 ± 2.86) and CD34 (91.2 ± 2.96) (**Figures 5D–I, 6**). In addition, cAVM-ECs were immunostained with negative EC markers, i.e., GFAP for astrocytes, SMA for smooth-muscle cells, and Tuj1 for neurons; however, no positive cells were observed. These results demonstrated that almost all the cultured cAVM-ECs purified using our protocol maintained the typical EC phenotype. In addition, the specific markers for ECs, vWF was also used to identify the origin of the cAVM-ECs (**Figure 7**). The cultured cAVM-ECs were functional, with the demonstrable ability to absorb Dil-Ac-LDL and form tube structures in 3D matrigel culture conditions. We found that Dil-Ac-LDL (labeled with red fluorescence) was in the cytoplasm of cAVM-ECs (**Figure 8A**) and the capillaries formed net-like structures (**Figure 8B**). However, the morphology of the isolated cAVM-ECs was not stable, and began to change into spindle-like cells after four generations of doubling (**Figure 9**).

### Utility of Human cAVM-ECs

Endothelial cells are useful tools to understand the biological characteristics of cerebral endothelium in conditions of neuroinflammatory, neurodegeneration, as well as neurovascular diseases. During vascular disease, ECs undergo cellular, morphogenetic, and phenotypic changes, accompanied with the appearance of angiogenic vasculature. Understanding EC dysfunction during cAVMs occurrence and development will be helpful to craft new therapies for the treatment of cAVMs.

The protocol we have established for isolating ECs from cAVMs can be also performed for other vascular diseases, such as cavernous hemangioma and aneurysm. For efficient EC isolation and purification, the vascular density level of CD31 expression and cAVM composition are important. This new and optimized method for isolating ECs will be useful for the study of cerebrovascular diseases.

## Materials

### Reagents

0.25% Trypsin-EDTA (Gibco, Cat. No. 25200072, United States);
FITC mouse anti-human CD31 antibody (BD, Cat. No. 557508, United States);
Rabbit polyclonal anti-CD31 (Abcam, Cat. No. Ab28364, United States);
Mouse monoclonal antibody of anti-CD34 (Abcam, Cat. No. Ab81289, United States);
Goat anti-vWF (Abcam, Cat. No. Ab 11713, United States);
Mouse anti-beta III Tubulin (Tuj1) (Abcam, Cat. No. Ab 78078, United States);
Rabbit anti-GFAP (Abcam, Cat. No. Ab 33922, United States);
Rabbit anti-alpha smooth-muscle Actin (α-SMA) (Abcam, Cat. No. Ab 5694, United States);
Donkey Anti-Rabbit IgG H&L (Alexa Fluor^®^ 647) (Abcam, Cat. No. Ab150075, United States);
Goat Anti-Mouse IgG H&L (Alexa Fluor^®^ 488) (Abcam, Cat. No. Ab150113, United States);
Donkey Anti-Goat IgG H&L (Alexa Fluor^®^ 488) (Abcam, Cat. No. Ab150137, United States);
Vectashield mounting medium containing 4′, 6-diamidino-2-phenylindole (DAPI) (Vector Laboratories, Cat. No. H1200, United States);
Endothelial cell medium (ECM-NG, ScienCell, Cat. No. 1001NG, United States);
Fetal Bovine Serum (FBS, ScienCell, Cat. No. 0025, United States);
Penicillin / Streptomycin solution (P/S, ScienCell, Cat. No. 0503, United States);
Endothelial Cell Growth Supplement (ECGS, ScienCell, Cat. No. 1052, United States);
Phosphate-buffered saline (PBS) solution (Life Technologies, Cat No. 10010023, United States);
Bovine serum albumin (BSA, Jackson Immunoresearch Laboratories, Cat. No. BAH640050, United States);
Dil-labeled acetylated LDL (Invitrogen, Cat No. L35353, United States);
Collagen type I (RD, Cat No. 3440-100-01, United States);
Heparin (Leo Pharma, Cat. No. 013192-02);
Cerebral AVM Tissues;
Matrigel (BD, Cat No. 354234, United States);
EC media;
FACS media;
LDL (Invitrogen, Cat No. L35353, United States)

### Equipment

Centrifuge suitable for 15-ml and 1.7-ml tubes
FACS (Millipore);
Surgical blades;
Surgical scissor
Disposable Petri dishes;
50-ml Tubes (Corning, Cat. No. 431720, United States);
15-ml Tubes (Corning, Cat. No. 430052, United States);
1.7-ml Tubes (Corning, Cat. No. 3622, United States);
1-ml Syringes;
Cell strainers (BD, 70um, Cat. No. 352350, United States);
FACS tubes with caps;
Bovine Serum Albumin (BSA) (Solarbio, Cat. No. A8020, China);
Matrigel (Corning, Cat. No. 354248, United States);
PBS (pH7.4) (Life Technologies, Cat. No. 10010023, United States)

### Reagent Setup

#### Patients and Tissues

Cerebral arteriovenous malformation samples were collected from eight patients who underwent surgical resection in the Department of Neurosurgery, Tian Tan Hospital, Beijing, China. Samples were collected between September 2015 and January 2016. Samples were collected from patients who had not previously received irradiation or embolization treatment. The Human Subject Review Committee of Tian Tan Hospital approved the experimental protocol. Tissue samples were collected immediately after surgical resection, which were placed in tubes with EC separation media and kept in the icebox for 6 h at most, before initiating the purification protocol.

#### Antibodies Used for Labeling and FACS

Titration of antibodies was crucial for isolating pure populations of cAVM-ECs. It was also recommended to titrate Ab that was EC-specific in the tissue of interest. We tested different titrations of Ab, and determined that 1:50 dilution of anti-CD31 was optimal for the isolation of cAVM-ECs. Human cAVM-ECs were sorted using FITC-labeled mouse anti-human CD31.

##### PBS/0.1%BSA

100 mg BSA was dissolved in 100 ml of PBS.

##### Collagen I (5 mg/ml)

100 mg Collagen I was dissolved in 20 mls of PBS.

##### Matrigel (80%wt/vol or 100%)

0.8 mg of Matrigel was dissolved in 0.2 ml EC culture medium.

#### EC Medium

Endothelial Cell Growth Supplement (ECGS, ScienCell, Cat. No. 1052, United States) was stored at -20°C. Once added to the media, it was stored at 4°C and protected from light. ECGS should be thawed only once and added to the media. ECGS was incubated at 37°C with occasional mixing until completely thawed before adding to the culture media in a BSC hood. The supplemented media should not be warmed using a 37°C water bath, and instead should be left to sit at room temperature protected from light before proceeding to cell culture. The reconstituted media was stable for 1 month.

#### FACS Medium

Cells were re-suspended in EC media supplemented with 0.1% BSA and were quickly sorted to prevent excessive cell death.

## Step-By-Step Procedure for Isolating and Purifying Ecs From Human cAVM Samples

### Preparation of Human AVM Samples

#### wTime Required: 6 h

(1) Phosphate-buffered saline solution was prepared with heparin and stored on ice. After surgical removal of cAVMs, the samples were immediately immersed in PBS solution with heparin.

#### Critical Step

To avoid blood clotting and reduce injury to ECs, samples were placed in PBS supplemented with heparin, and addition of heparin also increased the efficiency of enzyme digestion. To maintain optimal viability, the samples should be processed within ∼6 h of surgical incision.

### Preparation of Single-Cell Suspensions for cAVM-ECs Isolation

#### wTime Required: 7–14 day

(2) Separated vasculature structures from human cAVM samples. cAVM samples contained abnormal vessels, surrounding brain tissues, blood cells, and some electro-coagulation materials. To remove unwanted cells, AVM nidus samples were soaked in PBS solution with heparin, and brain and surrounding tissues were separated under a micro-dissecting instrument. The electro-coagulation materials were removed in a similar manner. For the removal of intravascular blood cells, we injected PBS with heparin into the lumen using a 1-ml syringe, to exclude blood cells retained in the vessel’s lumen (**Figures 2** Step 1, **3A,B**).

#### Critical Step

In order to avoid microbial contamination, the procedure should be carried out using sterile reagents and a laminar flow cabinet. The whole procedure was performed on ice.

(3) Cerebral arteriovenous malformations were cut into small cubes (**Figure 2** Step 2), and subsequently 0.25% Trypsin-EDTA was added. The tissues were further chopped and then incubated at 37°C with 5% CO_2_ for 15 min (**Figures 2** Steps 3, 4, **3C,D**).

#### Critical Step

Inefficient digestion of cAVMs with trypsin might cause cell clusters to not pass through cell filters and result in reduced cell yield. In order to avoid this, tissues should be gently pipetted up/down several times during the 15-min incubation.

(4) Neutralize Trypsin-EDTA with EC culture medium (please refer to solution 2:1). The digested cAVMs were then filtered twice using an additional 5 ml of EC culture media.

(5) The filtered cells were centrifuged at a speed of 1000 rpm for 5 min, then resuspended in EC culture media, and then centrifuged again. The cell pellets were then resuspended in fresh EC culture media (**Figure 2** Step 5).

(6) 6-well plates were coated with Collagen I (5 mg/ml). 2mls of Collagen I solution were added into wells and incubated under 37° C for 1 h. Then the Collagen I solution was removed and 6-well plates were washed three times with PBS. The cells were then seeded into 6-well plates. Large aggregates were removed from the culture media. The cells were incubated for 7–14 day until colonies were observed (**Figure 2** Step 6).

#### Critical Step

Collagen I could promote cell attachment and be reused three times at most.

### EC Purification

#### wTime Required: 6 h

(7) cAVM-ECs were cultured in ECM and harvested after 7–14 days. The cells were washed three times with PBS, and digested by 0.25% Trypsin-EDTA. After 5–10 min digestion, the cells solution was added to ECM to neutralize Trypsin-EDTA. Then the cells solution was centrifuged at a speed of 1000 rpm for 5 min. cAVM-ECs were washed once with blocking buffer [1% BSA in 1× PBS with Calcium and Magnesium] and then suspended in blocking buffer to a density of 0.5 × 10^5^ cells/ml at RT for 30 min. 1.0 μl of anti-human CD31 antibody (BD, Cat. No. 557508, United States) was then added to the cells and incubated for at least 30 min with gentle agitation. cAVM-ECs were kept protected from light and incubated on ice before being analyzed by FACS.

#### Critical Step

All procedures should be performed in the cell culture hood to maintain sterility. It was also recommended to perform anti-mycoplasma tests at the end of the protocol to be sure that the endothelial cells were mycoplasma free.

### EC Immunocytochemistry

#### wTime Required: 20 h

(8) cAVM-ECs were fixed in 4% paraformaldehyde for 20 min at RT. Cells were then perforated permeabilized for 20 min using 0.3% Triton in PBS, and later washed three times with PBS, and subsequently blocked in 5% goat serum and incubated at 4°C with the following primary antibodies: Rabbit polyclonal anti-CD31 (1:200, Abcam, Cat No. Ab28364, United States), Mouse monoclonal antibody of anti-CD34 (1:100, Abcam, Cat No. Ab81289, United States), and Goat anti-Von Willebrand factor (vWF) (1:100, Abcam, Cat. No. Ab 11713, United States). The slides were then incubated with the appropriate secondary antibody, Donkey Anti-Rabbit IgG H&L (Alexa Fluor^®^ 647) (1:200, Abcam, Cat No. Ab150075, United States), Goat Anti-Mouse IgG H&L (Alexa Fluor^®^ 488) (1:200, Abcam, Cat No. Ab150113, United States), and Donkey Anti-Goat IgG H&L (Alexa Fluor^®^ 488) (1:200, Abcam, Cat. No. Ab150137, United States) for 1 h. Immunostained cAVM-ECs were counted randomly using five microscope fields in a coverslip using a Nikon Eclipse inverted-fluorescence microscope.

### EC Dil-Ac-LDL Uptake

#### wTime Required: 6 h

(9) The uptake of acetylated LDL served as a useful marker for identifying ECs. cAVM-ECs were incubated with Dil-labeled acetylated LDL (Invitrogen, Catalog No. L35353, United States) at RM for 4 h. Then cAVM-ECs were visualized and imaged using a Nikon Eclipse inverted-fluorescence microscope.

### EC Capillary Tube-Like Structure Formation

#### wTime Required: 8 h

(10) To determine whether ECM-cultured cAVM-ECs retained angiogenic properties, cells were plated at a density of 0.4 × 10^5^ onto surfaces coated with matrigel to detect formation of capillary tube-like structures. Reduced growth factor matrigel (BD, Cat No.354234, United States) was thawed at 4°C overnight in a refrigerator. 200 μl of matrigel was added into a 48-well plate, which was incubated at 37°C and under 5% CO_2_ for 30 min. Then the cAVM-ECs were seeded into each well and cultured for 12 h.

### Anticipated Results

Using the protocol we developed, isolated cAVM-ECs grew into separate colonies, with morphologies of round borders and cobblestone appearance. Observed under a microscope, cAVM-ECs showed flat and ellipse shape in morphology when confluent (**Figure 5**). After FACS, the proportion of cAVM-ECs was about 1.5% percentage of the total cell population (**Figure 4**). All primary ECs established from cAVMs were viable for a long period and expressed specific cell receptors, such as CD31, CD34, and vWF. In addition, AVM-ECs were with the demonstrable ability to absorb Dil-Ac-LDL and form tube structures in 3D matrigel culture conditions (**Figure 8**). These results demonstrated that cultured cAVM-ECs using our protocol were functional ECs, which had common cellular characteristics of ECs. The isolated cAVM-ECs should be used before four passages, because they would *trans*-differentiate into mesenchymal-like cells after four generations of doubling.

### Statistical Analysis

All experimental data were presented as Mean ± SD, using GraphPad Prism 6 (United States).

## Ethics Statement

This study was carried out in accordance with the recommendations of Bei Jing Tian Hospital of guidelines, Bei Jing Tian Tan Hospital of committee’ with written informed consent from all subjects. All subjects gave written informed consent in accordance with the Declaration of Helsinki. The protocol was approved by the Bei Jing Tian Hospital of committee.

## Author Contributions

Y-LZ conceived and directed the project. QH designed the experiment and participated in the whole proceed, and wrote the manuscript. X-LC and LM participated in the discussion. T-TW and YH participated in the culture of cells.

## Conflict of Interest Statement

The authors declare that the research was conducted in the absence of any commercial or financial relationships that could be construed as a potential conflict of interest.
